# *Cmah*-dystrophin deficient *mdx* mice display an accelerated cardiac phenotype that is improved following peptide-PMO exon skipping treatment

**DOI:** 10.1093/hmg/ddy346

**Published:** 2018-10-02

**Authors:** Corinne A Betts, Graham McClorey, Richard Healicon, Suzan M Hammond, Raquel Manzano, Sofia Muses, Vicky Ball, Caroline Godfrey, Thomas M Merritt, Tirsa van Westering, Liz O’Donovan, Kim E Wells, Michael J Gait, Dominic J Wells, Damian Tyler, Matthew J Wood

**Affiliations:** 1 Department of Physiology, Anatomy and Genetics, University of Oxford, South Parks Road, Oxford, UK; 2 Department of Comparative Biomedical Sciences, Royal Veterinary College, Royal College Street, London, UK; 3 Clinical Biomanufacturing Facility, Nuffield Department of Clinical Medicine, University of Oxford, Old Road, Oxford, UK; 4 Medical Research Council, Laboratory of Molecular Biology, Francis Crick Avenue, Cambridge, UK

## Abstract

Duchenne muscular dystrophy (DMD) is caused by loss of dystrophin protein, leading to progressive muscle weakness and premature death due to respiratory and/or cardiac complications. Cardiac involvement is characterized by progressive dilated cardiomyopathy, decreased fractional shortening and metabolic dysfunction involving reduced metabolism of fatty acids—the major cardiac metabolic substrate. Several mouse models have been developed to study molecular and pathological consequences of dystrophin deficiency, but do not recapitulate all aspects of human disease pathology and exhibit a mild cardiac phenotype. Here we demonstrate that *Cmah* (cytidine monophosphate-sialic acid hydroxylase)-deficient *mdx* mice (*Cmah−/−;mdx)* have an accelerated cardiac phenotype compared to the established *mdx* model. *Cmah−/−;mdx* mice display earlier functional deterioration, specifically a reduction in right ventricle (RV) ejection fraction and stroke volume (SV) at 12 weeks of age and decreased left ventricle diastolic volume with subsequent reduced SV compared to *mdx* mice by 24 weeks. They further show earlier elevation of cardiac damage markers for fibrosis (*Ctgf*)*,* oxidative damage (*Nox4*) and haemodynamic load (*Nppa*)*.* Cardiac metabolic substrate requirement was assessed using hyperpolarized magnetic resonance spectroscopy indicating increased *in vivo* glycolytic flux in *Cmah−/−;mdx* mice. Early upregulation of mitochondrial genes (*Ucp3* and *Cpt1*) and downregulation of key glycolytic genes (*Pdk1, Pdk4, Ppara*), also denote disturbed cardiac metabolism and shift towards glucose utilization in *Cmah−/−;mdx* mice. Moreover, we show long-term treatment with peptide-conjugated exon skipping antisense oligonucleotides (20-week regimen), resulted in 20% cardiac dystrophin protein restoration and significantly improved RV cardiac function. Therefore, *Cmah−/−;mdx* mice represent an appropriate model for evaluating cardiac benefit of novel DMD therapeutics.

## Introduction

Duchenne muscular dystrophy (DMD) is caused by the loss of dystrophin protein due to genetic defects in the *DMD* gene (1). This results in rapidly progressing muscle wasting with subsequent early loss of ambulation (2) and death in the third or fourth decade of life. As the standard of care has improved for patients, predominantly through the use of corticosteroids to delay disease progression (3) and the use of assisted ventilation to preserve respiratory function (4), men are living longer and as such, secondary pathologies such as cardiomyopathy have become important factors to consider for disease management. Cardiomyopathy in DMD patients first presents with left ventricle (LV) dilation and hypertrophy accompanied by decreased fractional shortening and electrocardiogram (ECG) abnormalities (5–8). As the disease progresses, patients exhibit dilated cardiomyopathy, and by 20 years of age most DMD patients suffer from cardiac complications (5). Cardiomyopathy is a major contributor of death amongst DMD patients (9), and, therefore, multiple approaches have been implemented to treat the cardiac phenotype, including corticosteroids, beta-blockers, angiotensin converting enzyme (ACE) inhibitors and antimineralocorticoid diuretics (10).

The heart is unique in its requirement for metabolic substrate particularly compared to skeletal muscle. The heart predominantly relies on free fatty acids (FFAs) for ∼70% of its energy requirements and for the remainder on glucose reserves (11). When the heart undergoes a shift in metabolic substrate requirement, for instance an increase in glucose utilization, this is generally deemed pathological, as is the case in heart failure patients. Disruption of lipid handling in skeletal muscle of DMD patients is well characterized and includes the accumulation of cholesterol, sphingomyelin and triglycerides, as well as high levels of monosaturated fatty acid in damaged areas of muscle (12). As cardiac biopsies are not feasible, assessment of cardiac metabolic alterations is more difficult in patients. However, there is one study that utilized radioiodinated branched fatty acid (^123^I-BMIPP) for *in vivo* measurement of myocardial metabolism in DMD patients (13). Momose *et al.* showed a reduction in myocardial fatty acid metabolism in a substantial proportion of patients.

To understand the molecular and pathological effects of the loss of dystrophin protein, a number of mouse models of DMD are available, including the *mdx* mouse that has a premature termination codon in exon 23 that results in loss of the full-length dystrophin isoform (14). The cardiac phenotype in this model is well characterized, typified by right ventricle (RV) dysfunction preceding LV involvement; although it should be noted that generally LV function deteriorates prior to RV in DMD patients (15,16). Multiple studies also report altered proteomic profile of key metabolic proteins, in particular mitochondrial proteins associated with the electron transport chain and ATP synthesis (17,18). Further to this, alterations in metabolic flux in isolated *mdx* hearts was observed as early as 12 weeks of age (19), which is prior to obvious histological and functional changes (15,20). Specifically, *mdx* mouse hearts displayed a marked shift from long chain fatty acid to carbohydrate oxidation for energy requirements, a decrease in utilization of the pyruvate carboxylation pathway and enhanced glycolysis.

More recently, another DMD mouse model, the *Cmah−/−;mdx* mouse, has been generated (21). In this model, the cytidine monophosphate-sialic acid hydroxylase (*Cmah*) gene, which is also absent in humans, is deleted on the *mdx* background, resulting in a more severe phenotype representative of the human disease. This included advanced inflammation and fibrosis, reduced specific force in muscle, increased susceptibility to contraction/exercise induced damage and increased mortality rates. The characterization of this model did not report on cardiac disease progression, and given that other measures of disease were worsened, we hypothesized that this model may be more appropriate to assess cardiac defects than the *mdx* model. We therefore sought to assess cardiac function by magnetic resonance imaging (MRI) and cardiac metabolic flux *in vivo* using hyperpolarized magnetic resonance spectroscopy (MRS) technology. Additionally, we sought to assess the benefit of dystrophin restoration in this model utilizing a splice-switching approach. This model contains a premature termination codon at exon 23, therefore we target the removal of exon 23 so as to restore near full-length dystrophin protein. To achieve this we used cell-penetrating peptide conjugated morpholinos, specifically Pip6A-PMO, that have been used in previous studies in *mdx* mice to achieve robust dystrophin expression in skeletal muscle and in the heart (22,23).

## Results

### Marked cardiac functional changes and damage in *mdx* and *Cmah−/−;mdx* mice from 12 weeks of age

**Figure 1:**
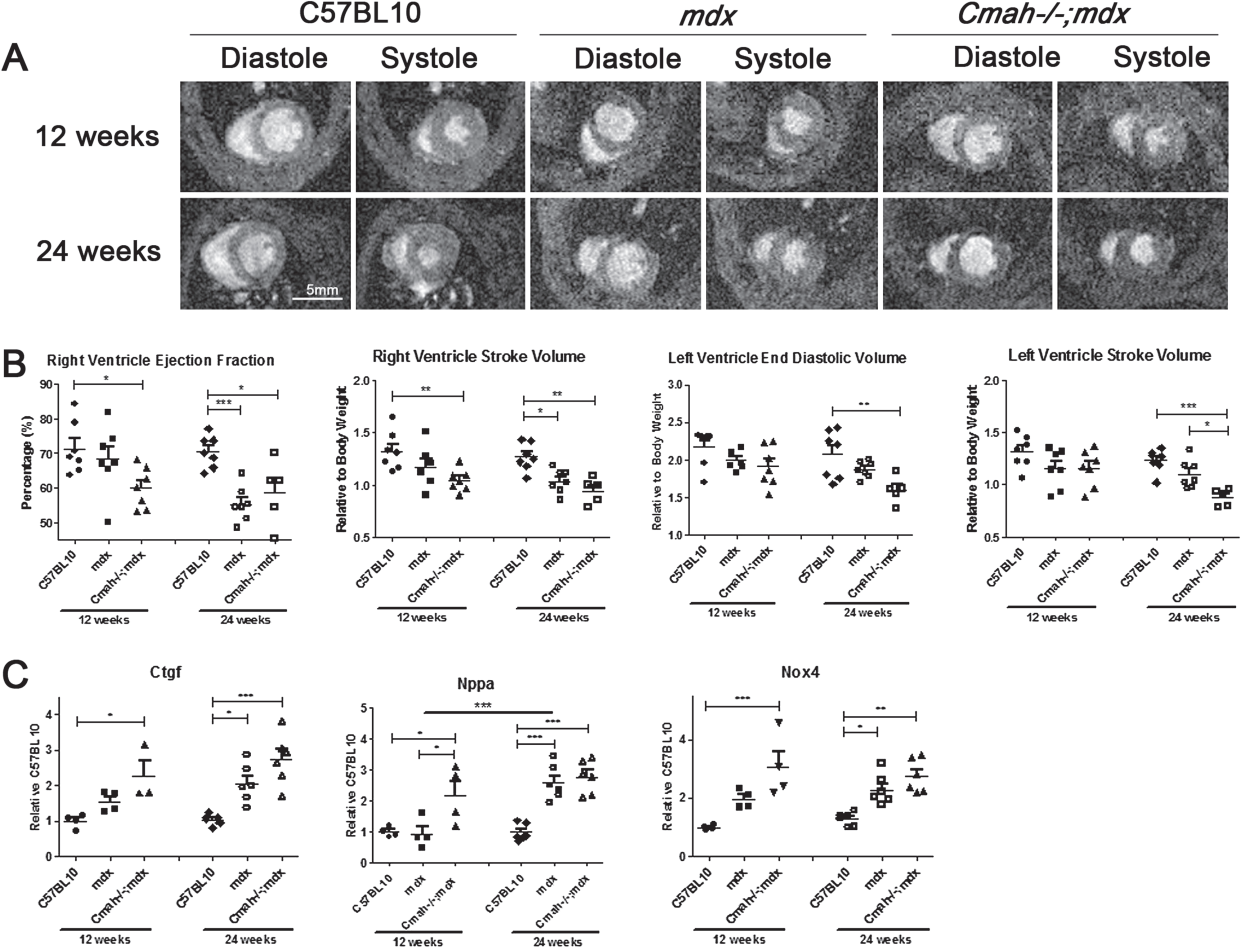
Cardiac function and pathology of *mdx* and *Cmah−/−;mdx* hearts compared to C57BL10 at 12 and 24 weeks of age. (**A**) Representative cine MRI images showing LV and RVs for heart during diastole and systole. (**B**) Cardiac function parameters, RV EF (%), RV SV, LV EDV and LV SV. (B) Quantitative real time (qRT)-PCR for the expression of fibrotic and injury markers namely *Ctgf*, *Nppa* and *Nox4* in heart tissue normalized to 12 week C57BL10. Data displayed as mean ± SEM. For cardiac function parameters *N* = 5–7, and qRT-PCR data *n* = 3–6. Significance calculated using two-way ANOVA, Tukey *post-hoc* test (^***^P < 0.001, ^**^P < 0.01, ^*^P < 0.05).

Mouse weights and cardiac function (cine-MRI) in C57LB10, *mdx* and *Cmah−/−;mdx* male mice were recorded at 12 and 24 weeks of age. At 12 weeks there was no significant difference in body weight (BW) between genotypes; however, at 24 weeks, BW was significantly raised in *Cmah−/−;mdx* mice compared to control and *mdx* mice, with *mdx* mice also significantly heavier than controls (Table 1). Multiple parameters including systolic and diastolic volumes, stroke volume (SV) and cardiac output (CO) were normalized to BW. Figure 1A shows representative stills for each cohort at diastole and systole for 12- and 24-week-old mice. As previously observed, there were no significant functional changes in the *mdx* cohort compared to C57BL10 mice at 12 weeks of age, with the exception of LV CO (Table 1) as previously reported (15,22). Interestingly, *Cmah−/−;mdx* mice exhibited reduced RV ejection fraction (RV EF; Fig. 1B), lower RV SV (Fig. 1B) with resultant lower CO (RV CO; Table 1) compared with C57BL10 at 12 weeks. At 24 weeks of age, the *mdx* mice revealed a smaller average LV mass and changes in RV function including reduced RV EF, lowered RV end-systolic volume (RV ESV) and RV SV, leading to reduced RV CO (Table 1). In addition to marked RV changes, *Cmah−/−;mdx* mice also exhibited reduced LV end-diastolic volume (LV EDV), LV SV and LV CO (Fig. 1B and Table 1). The heart rate (HR) of *Cmah−/−;mdx* mice was also significantly elevated. Further to functional abnormalities, *Cmah−/−;mdx* mice also displayed elevated gene expression of the fibrosis marker *Connective tissue growth factor* (*Ctgf*; Fig. 1C) at 12 and 24 weeks of age. Markers of cardiac damage/haemodynamic load [oxidative stress (NADPH oxidase 4; *Nox4*) and natriuretic peptide precursor A (*Nppa*)] (24,25) were also significantly upregulated in *Cmah−/−;mdx* mice at these time points. It is noteworthy to add that in *mdx* mice the expression of *Ctgf* and *Nppa* was only significantly elevated at 24 weeks of age. *Nox4* expression was elevated in *mdx* mice from 12 weeks of age but was significantly lower than the *Cmah−/−;mdx* cohort. This data is complemented by histology showing marked fibrosis at the LV and RV walls of *Cmah−/−;mdx* mouse hearts at 12 weeks of age compared to *mdx* and C57BL10 cohorts (Supplementary Material, Fig. S1).

**Table 1:** Cine MRI measurements from C57BL10, *mdx* and *Cmah−/−;mdx* mice at 12 and 24 weeks of age

Parameter	12 Weeks			24 Weeks		
	C57BL10	*mdx*	*Cmah−/−;mdx*	C57BL10	*mdx*	*Cmah−/−;mdx*
BW (g)	27.1 ± 0.5	28.3 ± 0.4	27.3 ± 0.5	32.3 ± 0.5	34.1 ± 0.5^*^	36.2 ± 0.5^****†^
HR (bpm)	482 ± 10	452 ± 10	466 ± 8	453 ± 20	445 ± 10	505 ± 10^*†^
Average LV mass/BW	3.95 ± 0.07	3.62 ± 0.09	3.57 ± 0.09^*^	4.14 ± 0.15	3.44 ± 0.06^****^	3.5 ± 0.2^***^
LV EF (%)	61 ± 4	58 ± 4	61 ± 3	60 ± 3	59 ± 3	55 ± 4
LV EDV/BW	2.18 ± 0.09	2.00 ± 0.05	1.9 ± 0.1	2.1 ± 0.1	1.87 ± 0.04	1.60 ± 0.08^**^
LV ESV/BW	0.9 ± 0.1	0.85 ± 0.08	0.76 ± 0.08	0.9 ± 0.1	0.78 ± 0.06	0.72 ± 0.09
LV SV/BW	1.31 ± 0.06	1.15 ± 0.07	1.15 ± 0.07	1.23 ± 0.04	1.10 ± 0.05	0.87 ± 0.04^***†^
LV CO/BW	0.63 ± 0.04	0.52 ± 0.04^*^	0.53 ± 0.03^*^	0.56 ± 0.03	0.49 ± 0.02	0.4 ± 0.2^*^
RV EF (%)	71 ± 3	68 ± 4	60 ± 2^*^	71 ± 2	55 ± 2^***^	59 ± 4^*^
RV EDV/BW	1.86 ± 0.05	1.72 ± 0.04	1.75 ± 0.09	1.80 ± 0.08	1.87 ± 0.05	1.61 ± 0.03†
RV ESV/BW	0.53 ± 0.05	0.55 ± 0.07	0.71 ± 0.07	0.54 ± 0.05	0.84 ± 0.04^**^	0.67 ± 0.08
RV SV/BW	1.32 ± 0.07	1.17 ± 0.07	1.04 ± 0.04^**^	1.27 ± 0.05	1.04 ± 0.04^*^	0.94 ± 0.05^**^
RV CO/BW	0.64 ± 0.05	0.53 ± 0.04	0.48 ± 0.02^**^	0.57 ± 0.03	0.46 ± 0.02^*^	0.48 ± 0.03

Data displayed as mean ± SEM. *N* = 5–7. Significance calculated using two-way ANOVA, Tukey *post-hoc* test (^****^P < 0.0001, ^***^P < 0.001, ^**^P < 0.01, ^*^P < 0.05). BW, body weight; HR, heart rate; LV, left ventricle; RV, right ventricle; EF, ejection fraction; ESV, end-systolic volume; EDV, end-diastolic volume; SV, stroke volume; CO, cardiac output.

**Figure 2:**
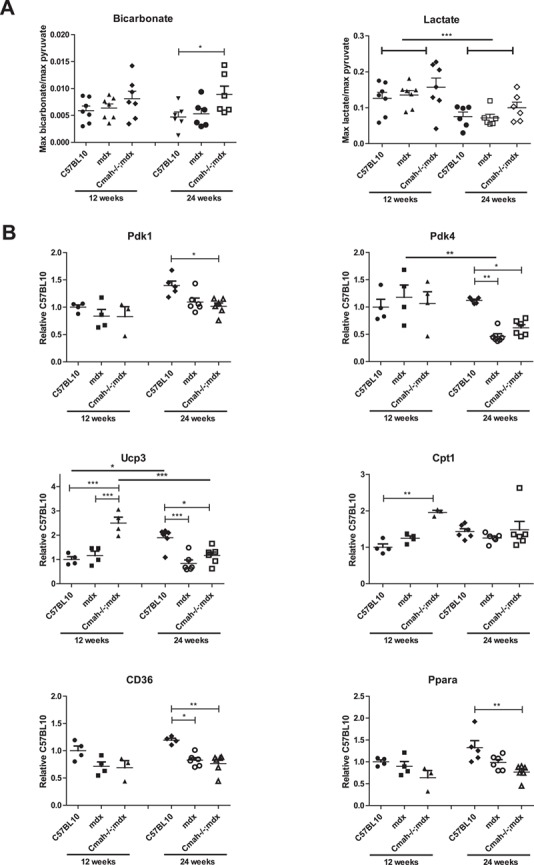
Metabolic profile of C57BL10, *mdx* and *Cmah−/−;mdx* hearts at 12 and 24 weeks of age-hyperpolarized MRS and gene expression analysis. (**A**) Bicarbonate and lactate production normalized to maximum pyruvate signal. Data displayed as mean ± SEM. *N* = 5–7. (**B**) Quantitative real-time (qRT)-PCR for the expression of *Pdk1*, *Pdk4*, *Ucp3*, *Cpt1*, *CD36* and *Ppara* in heart tissue normalized to 12-week C57BL10. Data displayed as mean ± SEM. *N* = 3–6. Statistical significance was determined using two-way ANOVA, Tukey *post-hoc* test (^****^P < 0.0001, ^***^P < 0.001, ^**^P < 0.01. ^*^P < 0.05).

### In vivo metabolic perturbations in *Cmah−/−;mdx* mice at 24 weeks

Hyperpolarized MRS was used to determine metabolic substrate allocation by measuring ^13^C labelled pyruvate flux through pyruvate dehydrogenase (PDH) and the citric acid cycle (26). This was measured in the hearts of 12 and 24 week old *mdx* and *Cmah−/−;mdx* male mice compared to C57LB10 controls. The rate of bicarbonate production at 24 weeks was significantly raised in *Cmah−/−;mdx* mice compared to the C57BL10 (Fig. 2A), indicating increased pyruvate flux through PDH. Although lactate levels were unchanged by genotype, age had a highly significant effect, with lactate production declining from 12 to 24 weeks of age across all genotypes. No statistically significant differences in alanine production were observed between any of the test groups at either time points (Supplementary Material, Fig. S2).

**Figure 3:**
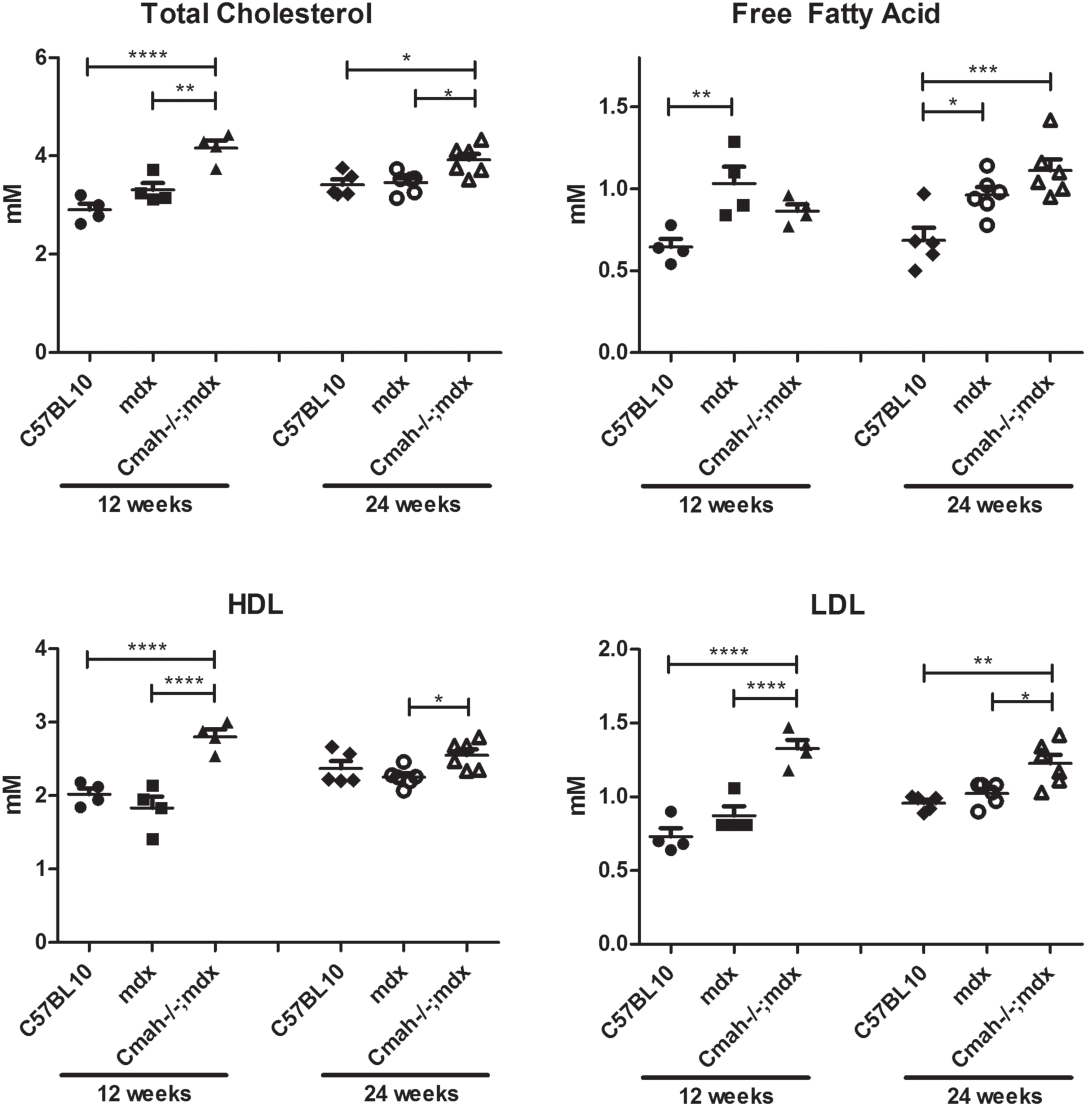
Biochemical data from plasma samples of C57BL10, *mdx* and *Cmah−/−;mdx* mice at 12 and 24 weeks. Graphs showing total cholesterol, FFA, HDL and LDL measurements. Data displayed as mean ± SEM. *N* = 4–6. Statistical significance was determined using two-way ANOVA, Tukey *post-hoc* test (^****^P < 0.0001, ^***^P < 0.001, ^**^P < 0.01, ^*^P < 0.05).

The expression levels of key metabolic genes involved in glucose and fatty acid metabolism were also altered. PDH lipoamide kinase isozymes 1 and 4 (*Pdk1* and *Pdk4*), were significantly downregulated in the *Cmah−/−;mdx* mouse cohort at 24 weeks of age (Fig. 2B; also downregulated in *mdx* for *Pdk4*). These kinases act to prevent the conversion of pyruvate from glucose, and thus a decrease in gene expression would suggest a shift towards glucose metabolism (27). Mitochondrial uncoupling protein 3 (*Ucp3*) is involved in the transfer of anions across the mitochondrial membrane in order to protect against oxidative stress (28). It is markedly upregulated at 12 weeks in *Cmah−/−;mdx* mice, suggesting a spike in oxidative stress. Carnitine palmitoyltransferase 1 (*Cpt1*) is a mitochondrial enzyme involved in fatty acid metabolism. *Cpt1* expression was unexpectedly upregulated in *Cmah−/−;mdx* mice at 12 weeks, however, it could be a compensatory mechanism attempting to catabolize fatty acids. Another important membrane protein, cluster of differentiation 36 (*CD36*), allows the importation of fatty acids and is significantly downregulated in both the *mdx* and *Cmah−/−;mdx* mice cohorts at 24 weeks. Peroxisome proliferator-activated receptor alpha (*Ppara*) is a key transcription factor that regulates lipid metabolism and is also significantly downregulated at 24 weeks of age. The downregulation of both *CD36* and *Ppara* support the hypothesis for a reduction in fatty acid metabolism. Indeed, circulating levels of total cholesterol, high-density lipoproteins (HDLs) and low-density lipoproteins (LDLs) were all elevated in *Cmah−/−;mdx* mice compared to C57BL10 and *mdx* cohorts, suggesting that these lipid reserves are not being utilized (Fig. 3 and Supplementary Material, Table S1). FFA levels were significantly raised in *mdx* mice at 12 weeks of age and in both *mdx* and *Cmah−/−;mdx* mice at 24 weeks.

**Figure 4:**
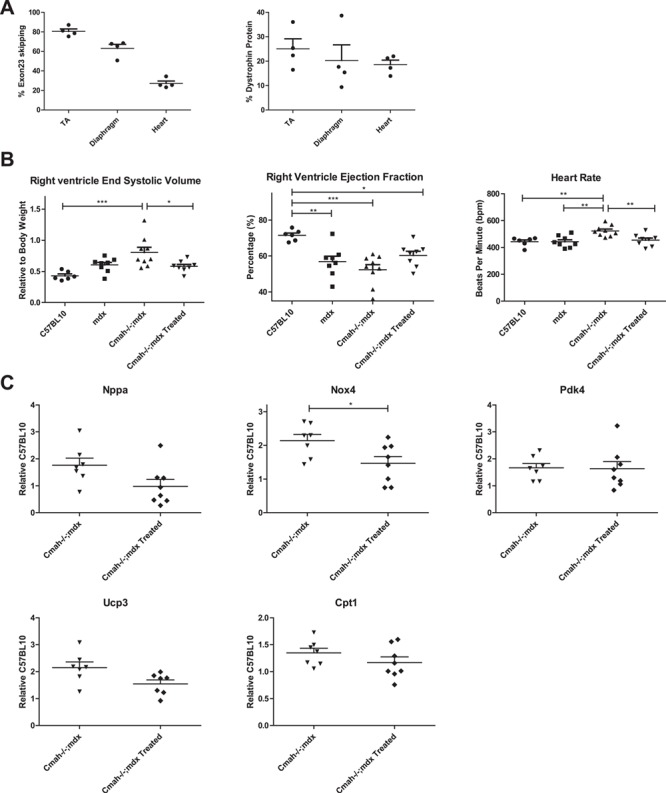
PPMO treatment restores dystrophin and improves RV function and pathology in *Cmah−/−;mdx* hearts at 8 months of age. (**A**) Quantification of exon 23-skipped transcripts (RT-qPCR) and dystrophin protein restoration in TA, diaphragm and heart following Pip6A-PMO treatment (*n* = 4). (**B**) Cardiac function parameters were measured by cine-MRI. Graphs indicating RV ESV, RV EF and HR shown. (**C**) RT-qPCR for the expression of injury and metabolic markers namely *Nox4*, *Nppa*, *Ucp3*, *Pdk1* and *Pdk4* in heart tissue normalized to C57BL10. Data displayed as mean ± SEM. For cardiac function parameters significance calculated using one-way ANOVA, Tukey *post-hoc* test. For RT-qPCR data significance was determined using Student’s *t*-test (^***^P < 0.001, ^**^P < 0.01^*^P < 0.05).

### Chronic PPMO treatment moderately improves RV function in *Cmah−/−;mdx*

To determine the treatment benefit of an exon-skipping approach to restore dystrophin in dystrophic hearts, 12-week-old *Cmah−/−;mdx* male mice (*n* = 7) were administered 10 × 10 mg/kg Pip6A-PMO, phosphorodiamidate Morpholino oligomer (Pip-PMO) fortnightly over a 20-week period. Approximately, 18% dystrophin protein restoration was detected in the heart by western blot, concurrent with moderate exon 23 skipping (Fig. 4A; Supplementary Material, Fig. S3). Immunohistochemistry also confirmed correct localization of dystrophin to the sarcolemma and widespread dystrophin positive fibres throughout the heart (Supplementary Material, Fig. S4).

Cardiac function in C57LB10, *mdx*, *Cmah−/−;mdx* and *Cmah−/−;mdx* treated mice was recorded using cine-MRI at 8 months of age. There was no significant difference in BW between genotypes (Table 2); however, the HR in *Cmah−/−;mdx* cohort was significantly raised compared to all other cohorts, suggesting compensatory activation of the sympathetic pathway (Fig. 4B). In addition, the RV ESV was markedly larger than the control cohort, resulting in decreased RV EF. However, Pip-PMO treatment restored HR and RV ESV to control levels. Interestingly, gene expression analysis of key metabolic factors at 36 weeks, indicate no significant difference between *Cmah−/−;mdx* and *Cmah−/−;mdx* treated cohorts. However, *Nppa* (trending) and *Nox4* were significantly downregulated in treated *Cmah−/−;mdx* cohorts indicating a reduction in damage and oxidative species (Fig. 4C). However, it should be noted that histological data shows marked fibrosis in the hearts of treated *Cmah−/−;mdx, mdx* and *Cmah−/−;mdx* mouse hearts at 8 months of age (Supplementary Material, Fig. S5).

**Table 2:** Cine MRI measurements from C57BL10, *mdx, Cmah−/−;mdx* and *Cmah−/−;mdx* treated mice at 8 months of age

Parameter	C57BL10	*mdx*	*Cmah−/−;mdx*	*Cmah−/−;mdx* Treated
BW (g)	35.00	38.00	38.33	38.25
HR (bpm)	442.62 ± 13.7	442.41 ± 15.2	523.1 ± 13.4^**^//	454.27 ± 15.8 ++
Average LV mass/BW	3.48 ± 0.15	2.93 ± 0.09 #	3.61 ± 0.21 /	3.51 ± 0. 09 /
LV EF (%)	63 ± 2.8	60 ± 3.8	54 ± 4.4	55 ± 4.3
LV EDV/BW	1.73 ± 0.11	1.37 ± 0.08	1.69 ± 0.18	1.69 ± 0.05
LV ESV/BW	0.65 ± 0.07	0.56 ± 0.06	0.83 ± 0.19	0.78 ± 0.09
LV SV/BW	1.09 ± 0.07	0.82 ± 0.06^*^	0.86 ± 0.06	0.91 ± 0.05
LV CO/BW	0.48 ± 0.04	0.36 ±^*^	0.45 ± 0.03	0.41 ± 0.02
RV EF (%)	72 ± 1.2	57 ± 3^**^	52 ± 2.8^***^	60 ± 2.3^*^
RV EDV/BW	1.51 ± 0.09	1.41 ± 0.07	1.67 ± 0.01	1.47 ± 0.03
RV ESV/BW	0.43 ± 0.03	0.60 ± 0.04	0.81 ± 0.08^***^	0.58 ± 0.03 +
RV SV/BW	1.08 ± 0.07	0.81 ± 0.06^*^	0.87 ± 0.06	0.89 ± 0.04
RV CO/BW	0.48	0.36^*^	0.45 /	0.40

Data displayed as mean ± SEM. *N* = 6–9. Significance calculated using one-way ANOVA, Tukey *post-hoc* test (^***^P < 0.001, ^**^P < 0.01, ^*^P < 0.05). BW, body weight; HR, heart rate; LV, left ventricle; RV, EF, ejection fraction; right ventricle; ESV, end-systolic volume; EDV, end-diastolic volume; SV, stroke volume; CO, cardiac output.

**Figure 5:**
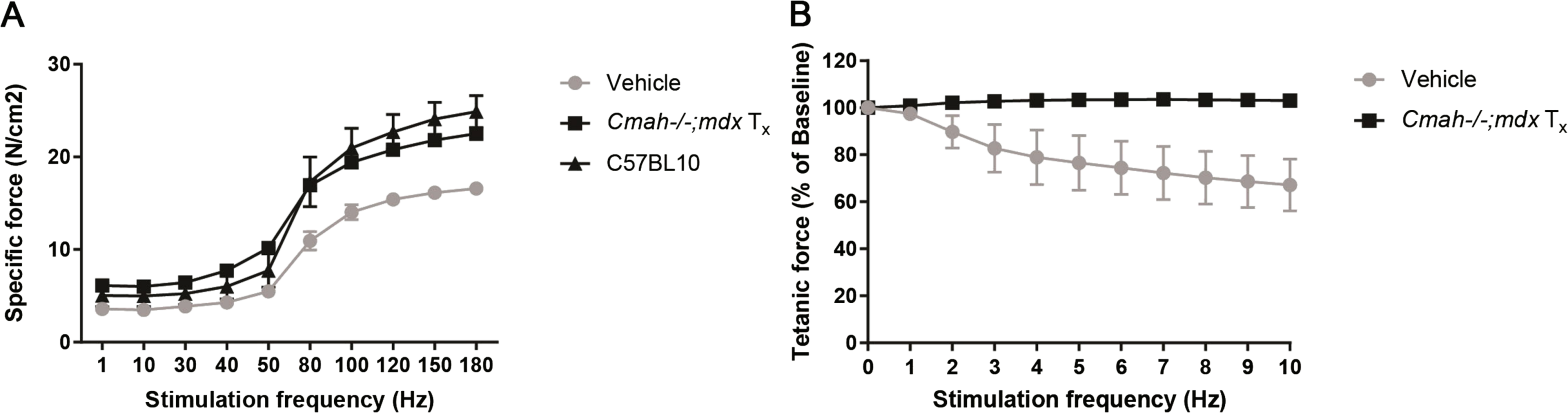
PPMO treatment improves muscle function in untreated and treated *Cmah−/−;mdx* mice. (**A**) Specific force–frequency relationship for untreated *Cmah−/−;mdx* (*n* = 6)*,* chronically Pip6a-PMO treated *Cmah−/−;mdx* (*n* = 8), and C57BL10 mice (*n* = 12). Treatment with Pip6a-PMO significantly increased specific force in the *Cmah−/−;mdx* mice (*P* < 0.001). (**B**) Force drop associated with eccentric exercise in untreated *Cmah−/−;mdx* and chronically Pip6a-PMO treated *Cmah−/−;mdx*. C57BL10 mice not included as their plot overlays the result for the chronically Pip6a-PMO treated *Cmah−/−;mdx* mice. The *Cmah−/−;mdx* mice show a significant force drop with eccentric exercise that is prevented by Pip6a-PMO treatment (*P* < 0.001). Pip6a-PMO treated *Cmah−/−;mdx* and C57BL10 mice are not significantly different.

### Chronic PPMO treatment induces robust dystrophin expression and improves and protects muscle function

Whilst the primary focus of this study was to evaluate cardiac function in this model, we also sought to determine if Pip-PMO mediated benefit to muscle function could be conferred by assessing resistance to eccentric contraction-induced damage in treated and untreated littermate controls (Fig. 5A). Untreated *Cmah−/−;mdx* mice exhibited a significant final drop in force of 33% compared with initial baseline force (*P* < 0.001), although this was not as pronounced as levels observed for *mdx* mice of ∼60% observed in previous studies (29). Administration of Pip-PMO prevented this force drop with maximal force production maintained throughout the eccentric contraction protocol, similar to wild-type C57BL10 mice (not shown). Specific isometric force was also significantly improved (*P* < 0.001) following Pip-PMO treatment with a 71% improvement towards wild-type levels compared to untreated *Cmah−/−;mdx* controls (Fig. 5B). Robust dystrophin protein restoration was detected in tibialis anterior (TA), diaphragm and heart, concurrent with high levels of exon 23 skipping, which is correlated to relative levels of correction in the different tissues (Fig. 4A). Immunohistochemistry also confirmed correct localization of dystrophin to the sarcolemma and widespread dystrophin positive fibres throughout quadriceps and diaphragm (Supplementary Material, Fig. S4). Muscle hypertrophy was also assessed with physiological cross-sectional area of the TA significantly reduced in Pip-PMO treated animals, indicative of reduced muscle hypertrophy (Supplementary Material, Table S2).

## Discussion

The predominant aim of this study was to evaluate cardiac dysfunction and metabolic profile in *Cmah−/−;mdx* and *mdx* mice to determine to what extent these models recapitulate the cardiac dystrophic pathology in DMD. Furthermore, we sought to determine if Pip-PMO-mediated exon skipping could confer functional benefit in the *Cmah−/−;mdx* model.

We show significant changes in functional cardiac parameters in the *Cmah−/−;mdx* model compared with C57BL10. We report alterations in multiple RV parameters as early as 12 weeks of age and prominent LV involvement by 24 weeks, which is markedly earlier then the *mdx* model (15,23). RV involvement does not generally precede LV dysfunction in patients, though it should be noted that DMD patients undergo rapid respiratory decline, and therefore ventilatory intervention is administered early. It is believed this improves pulmonary hypertension thus compensating for RV dysfunction (30,31). Indeed the decline in RV function in DMD patients does correlate with a reduction in respiratory function (32). This could account for marked disparity observed between DMD patients and the *Cmah−/−;mdx* and *mdx* models that do not receive ventilatory assistance. However, most importantly, *Cmah−/−;mdx* mice do exhibit a much earlier and more pronounced cardiac phenotype compared to *mdx*. These functional events also correlate with the elevation of multiple markers of pathology and damage. We are also the first to describe functional cardiac metabolic alterations in the *Cmah−/−;mdx* mouse, which again correlate with the downregulation of key fatty acid-handling genes and glucose pathway mediators. These data support previous reports in *mdx* mice (19) that show the dystrophic heart has a preference for glucose utilization, which is an important pathological indicator. Although we did observe significant changes in metabolic flux in *Cmah−/−;mdx* mice, we did not detect alterations in *mdx* hearts at 12 weeks of age as previously reported (19). It is important to note that our measurements were performed *in vivo*, but Khairallah *et al.* experiments were performed *ex vivo*. It is likely the differences underlying the *in vivo* and *ex vivo* approaches are due to systemic compensatory mechanisms attempting to reinstate a homeostatic state, and it is these events that are masking the underlying pathology of the *mdx* heart. However, most importantly we were able to detect significant changes in the *Cmah−/−;mdx* heart, indicating this is a more appropriate model for studying early metabolic perturbations. In short, the *Cmah−/−;mdx* model exhibits an earlier cardiac phenotype as determined by deterioration in heart function, cardiac metabolic perturbations and progressed pathology from 12 weeks of age, prior to alterations in *mdx* heart. It should be noted that by 24 weeks of age, the *mdx* heart also deteriorates, and many functional and pathological changes are evident.

Similarly to previous studies in the *mdx* mouse model (29), we observed marked dystrophin restoration in heart of *Cmah−/−;mdx* mice following administration of Pip-PMO targeting removal of exon 23 from the *Dmd* transcript. This restoration led to moderate improvement in some RV cardiac parameters and HR, but no improvement in metabolic gene expression levels. As such, PDH flux levels were not reported for the treated samples. These modest changes may be due to several factors: it may be that the treatment started too late and we missed the therapeutic window, that there is greater intraspecies variation within each cohort in older mice (evidenced by the higher SEM for some *Cmah−/−;mdx* cardiac parameters at 36 weeks) or that the interspecies variation between the *Cmah−/−;mdx* and *mdx* cohorts is diminished (the cardiac *mdx* phenotype deteriorates by 24 weeks exhibiting a similar phenotype to *Cmah−/−;mdx* mice). We also observed marked dystrophin restoration in skeletal muscle, and these levels were sufficient to induce a significant improvement in muscle physiology measures, with ∼70% correction towards wild-type levels for muscle specific force and complete protection against muscle force drop following eccentric contraction protocol. Interestingly, for the *Cmah−/−;mdx* vehicle group a number of mice did not exhibit as great a force drop following the eccentric protocol as observed previously for *mdx*, which suggests that they may not be as good a model as the *mdx* for assessing treatments targeted to skeletal muscle.

Assessment of metabolic pathways in the *mdx* model have utilized *ex vivo* approaches that demonstrated a clear shift in metabolic substrates towards glycolysis from as early as 12 weeks. In contrast, utilizing *in vivo* hyperpolarized MRS, we observed only a small shift towards glycolytic flux in *Cmah−/−mdx*, but not in *mdx*, even by 24 weeks of age. This suggests that despite a cardiac functional deficit in these mouse models, alterations in metabolic function are less evident and *in vivo* hyper-polarized MRS may not represent an appropriate measure for treatment benefit in these models.

Previous studies in *mdx* mice demonstrate onset of cardiomyopathy from 6 months. Our data presented here suggest that cardiac dysfunction in the *Cmah/mdx* model is not only established earlier (by 12 weeks) but also displays greater severity, and as such may be a more appropriate model to assess cardiac function. Ultimately, this research shows that the *Cmah−/−;mdx* mouse is an appropriate model to use if an early dystrophic heart is required for study.

## Materials and Methods

All experiments were performed at the University of Oxford, except for the muscle physiology experiments, which were conducted at the Royal Veterinary College London. All experiments were performed under the authorization of the UK Home Office. Mice were housed in individually ventilated caging systems (IVCs), with access to food and water *ad libitum*. All animals used were male.

### 

#### [1-^13^C]pyruvate hyperpolarized MRS and MRS analysis

The hyperpolarized MRS protocol was performed as previously described (26). In brief, mice were anaesthetized using 4% isoflurane for induction and then maintained at 1.5% isoflurane for the duration of the experiment. An intravenous line was established in the tail vein for the administration of hyperpolarized solutions. Mice were weighed and then placed supine into a custom-made cradle and ECG leads were inserted in the forepaws. The cradle was then inserted into the horizontal bore of a 7T MRI scanner connected to a direct drive console (Varian, Inc. Palo Alto, California, US). After preliminary axial scans to localize the heart, 0.15 ml of [1-^13^C]pyruvate, hyperpolarized as described^24^, was injected over 10 s and cardiac gated ^13^C MR spectra acquired every second for 60 s (10 mm radius ^13^C RF coil). MRS spectra were analysed using the AMARES algorithm in jMRUI software^25^, employing the maximum metabolite signal normalized to the maximum pyruvate signal to correct for variation in sample polarization and injection rate.

#### Cine MRI protocol and data analysis

Mice underwent Cine MRI as previously described (22). In brief, mice were anaesthetized using 4% isoflurane for induction and then maintained at 1.5% isoflurane for the duration of the experiment. Mice were weighed and placed supine in a custom-made cradle. ECG leads were inserted into the forearms and a respiratory lead taped across the chest of the mouse. The cradle was inserted into the vertical-bore of an 11.7 T MR system (Magnex Scientific, Yarnton, Oxford, UK) containing a 40 mm birdcage RF coil (Rapid Biomedical, Rimpar, Germany). Images were taken using a Bruker console running Paravision 2.1.1 (Bruker Medical, Coventry, UK). Preliminary axial scans ensured correct positioning of the animal within the coil. The LV and RVs were imaged by taking a contiguous stack of cine images in 1 mm increments and images were analysed using ImageJ software (NIH Image, Bethesda). The epicardial and endocardial borders at end-diastole and end-systole were outlined using the ImageJ free-hand tool. These measurements were used to calculate multiple cardiac structure and function parameters.

Mice were sacrificed by CO_2_ inhalation followed by cervical dislocation. Blood, heart, TA and diaphragm tissue samples were collected and snap frozen and stored at −80°C.

#### RNA extraction, cDNA synthesis and qPCR

Total RNA was extracted using TRIzol reagent (Invitrogen, Paisley, UK) as described in the manufacturer’s instructions. 1 ug of RNA was reverse transcribed using a High Capacity cDNA Synthesis kit (ThermoFisher, Paisley, UK). Diluted cDNA was run using gene specific primers sets (IDT; see Supplementary Material, Table S3 for Assay IDs) and TaqMan probe set (Integrated DNA Technologies, Leuven, Belgium) on the StepOne Plus Real-Time PCR system (Applied Biosystems, Paisley, UK). Samples were quantified using the Pfaffl method by attaining the CT values for each reaction and then normalizing relative to the house-keeping gene, Ywhaz.

Levels of *Dmd* exon 23 skipping were determined by multiplex qPCR of FAM-labelled primers spanning Exon 20–21 (Assay Mm.PT.47.9564450, Integrated DNA Technologies) and HEX-labelled primers spanning Exon 23–24 (Mm.PT.47.7668824, Integrated DNA Technologies). The percentage of *Dmd* transcripts containing exon 23 was determined by normalizing exon 23–24 amplification levels to exon 20–21 levels.

#### Plasma and liver biochemistry

Blood was collected from the jugular vein of mice immediately after sacrifice in plasma tubes (Sarstedt) and sent to the MRC Harwell Mary Lyon Centre (Oxfordshire, UK) for biochemical analysis.

#### Masson trichrome staining

A total of 8 μm transverse sections of heart were cut and mounted on slides. Slides were sent to the Dunn School of Pathology, University of Oxford, for staining. Images were captured with a DM IRB Leica upright microscope (Zeiss monochrome camera) and AxioVision Rel. 4.8 software.

#### P-PMO synthesis, preparation and administration

Pip6a was synthesized by standard solid phase Fmoc chemistry and purified by HPLC, as previously described (22, 23). The PMO sequence (5′-GGCCAAACCTCGGCTTACCTGAAAT-3′) was purchased from Gene Tools LLC. Pip6a was conjugated to PMO through an amide linkage at the 3′ end of the PMO, followed by purification by HPLC. The final product was analysed by MALDI-TOF MS and HPLC.

For chronic PPMO treatment, 12-week-old male *Cmah*−/−*mdx* mice were administered 10 intravenous tail-vein injections of Pip6a-PMO (12.5 mg/kg) at 2-week intervals. Littermate mice were used as untreated controls.

#### Muscle physiology

Two weeks after the last Pip6a-PMO injection, muscle function was assessed using the right TA muscle. Mice were surgically prepared and analysed as previously described (29).

Briefly, optimal muscle length (Lo) was determined by increasing muscle length until the maximal twitch force was achieved. For force–frequency relationship, TA muscles were stimulated at different frequencies, delivered 1 min apart (1, 10, 30, 40, 50, 80, 100, 120, 150 and 180 Hz). Muscle fibre physiological cross-sectional area (CSA in cm^2^) was determined as previously described (29) and specific isometric force (N/cm^2^) was calculated by dividing the absolute force (N) at each stimulation frequency by TA muscle physiological cross-sectional area.

Following the force/frequency testing the muscle was tested with an eccentric contraction protocol. The TA muscle was stimulated at 120 Hz for 500 ms before lengthening the muscle by 10% of the Lo at a velocity of 0.5 Lo s-1 for a further 200 ms. Between each contraction a 2 min rest period was permitted to avoid muscle fatigue. A total of 10 eccentric contractions were performed on each mouse. After each eccentric contraction, the maximum isometric force was measured and expressed as a percentage of the initial maximum isometric force achieved at the start of the protocol, prior to the first eccentric contraction. Statistical analysis for the force–frequency and eccentric contraction studies was measured by a repeated measure two-way ANOVA followed by a Tukey’s *post-hoc* comparison. Statistical significance was defined as a value of *P* < 0.05.

### Dystrophin protein extraction and western blot analysis

For dystrophin protein quantification, transverse cryosections (8 μm thick) were lysed in buffer containing 75 mm/l Tris–HCl (pH 6.5), 10% sodium dodecyl sulphate, 5% 2-mercaptoethanol, centrifuged at 13 000 rpm for 10 min and supernatant was collected and heated at 100°C for 3 min. Proteins were resolved on a 3–8% Tris–Acetate gel (Invitrogen) and transferred to PVDF membranes for 100 minutes at 30 V (Millipore, Hertfordshire, UK). Subsequently, membranes were probed with monoclonal anti-dystrophin (1:200, NCL-DYS1, Novocastra, Milton Keynes, UK) and anti-vinculin (loading control, 1:100 000, hVIN-1, Sigma) antibodies and detected with secondary antibody IRDye 800CW goat anti-mouse (LiCOR, Cambridge, UK). Fluorescence was recorded using the Odyssey imaging system. To quantify dystrophin expression, the ratio between dystrophin and vinculin signals was plotted and referred to the ratio of C57BL10 protein standard dilutions on each gel, set as 100%.

### Dystrophin Immunohistochemistry analysis

A total of 8 μm transverse sections of quadriceps, diaphragm and heart tissues were cut and mounted on slides. Unfixed sections were blocked for 2 h in 20% foetal bovine serum, 20% goat serum solution and double stained for 2 h with antibodies for dystrophin (Abcam, Cambridge, UK, ab15277) and laminin α2 (Sigma, Dorset, UK, L0663). Primary antibodies were detected with Alexa 594 and 488, respectively, and mounted in Dako Fluorescent Mounting Medium. Images were captured with a DM IRB Leica upright microscope (Zeiss monochrome camera) and AxioVision Rel. 4.8 software.

### Statistical analysis

All reported values are mean ± standard error of the mean (SEM). When two groups were compared, a Student’s *t*-test was utilized. When multiple groups were analysed, Two-way ANOVA and One-way ANOVA with Tukey *post-hoc* method was employed. Graphs were drawn using GraphPad Prism 5 software (GraphPad Software Inc, USA). Grubbs test was performed to exclude outliers.

## Supplementary Material

Supplementary Data
