# Supplementary material for: Cmah-dystrophin deficient mdx mice display an accelerated cardiac phenotype that is improved following peptide-PMO exon skipping treatment
Source: Hum Mol Genet. 2018 Oct 2;28(3):396–406. doi: 10.1093/hmg/ddy346 (PMC6337703; doi:10.1093/hmg/ddy346)
Supplement: Supplementary Data [file ddy346_supp_data.doc]

**
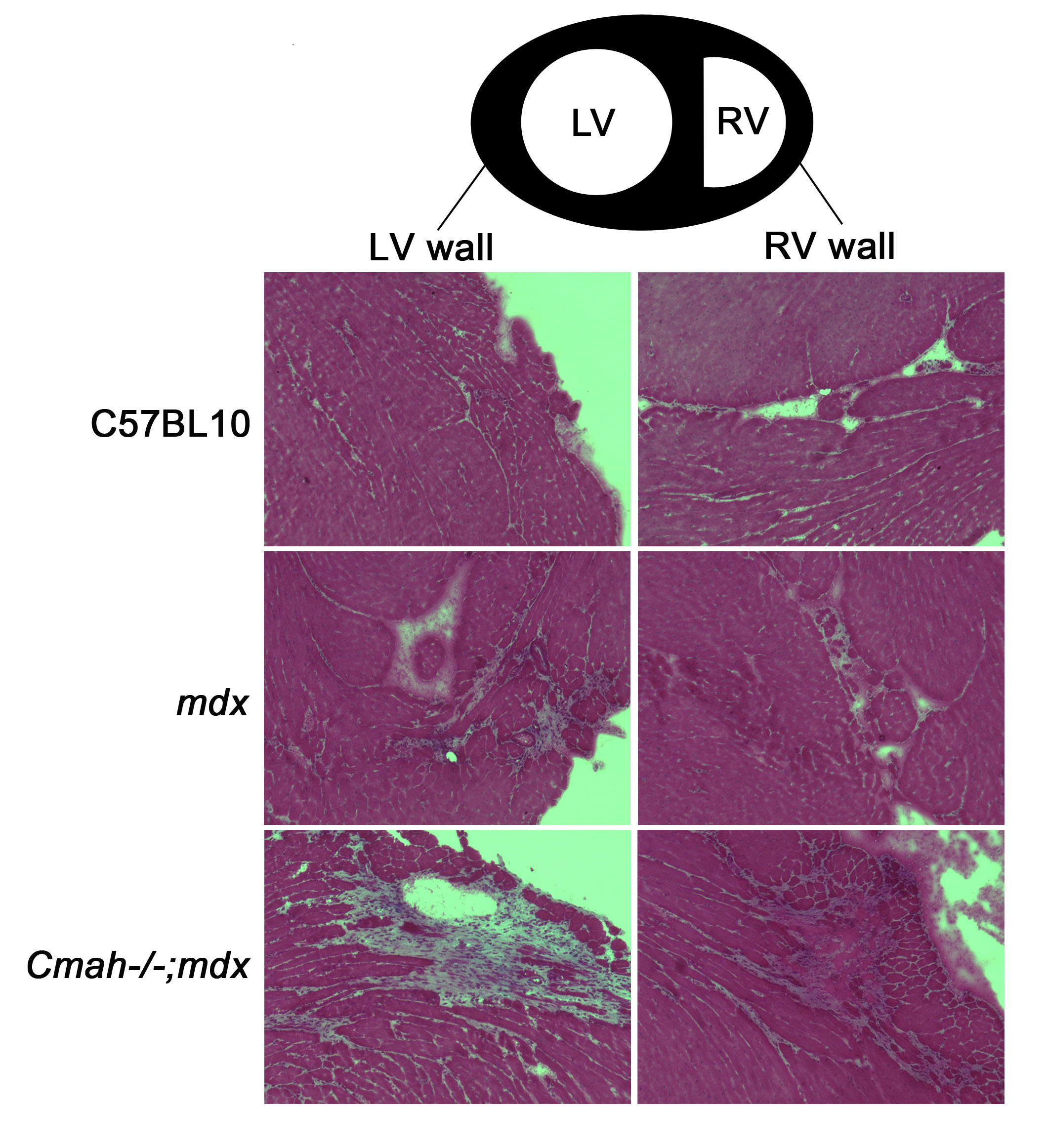
Supp Figure 1. Masson trichrome staining of C57BL10, *mdx* and *Cmah-/-;mdx* hearts at 12 weeks of age.** Representative images showing fibrosis on left ventricle (LV) wall and right ventricle (RV) wall in *Cmah-/-;mdx* hearts.

**
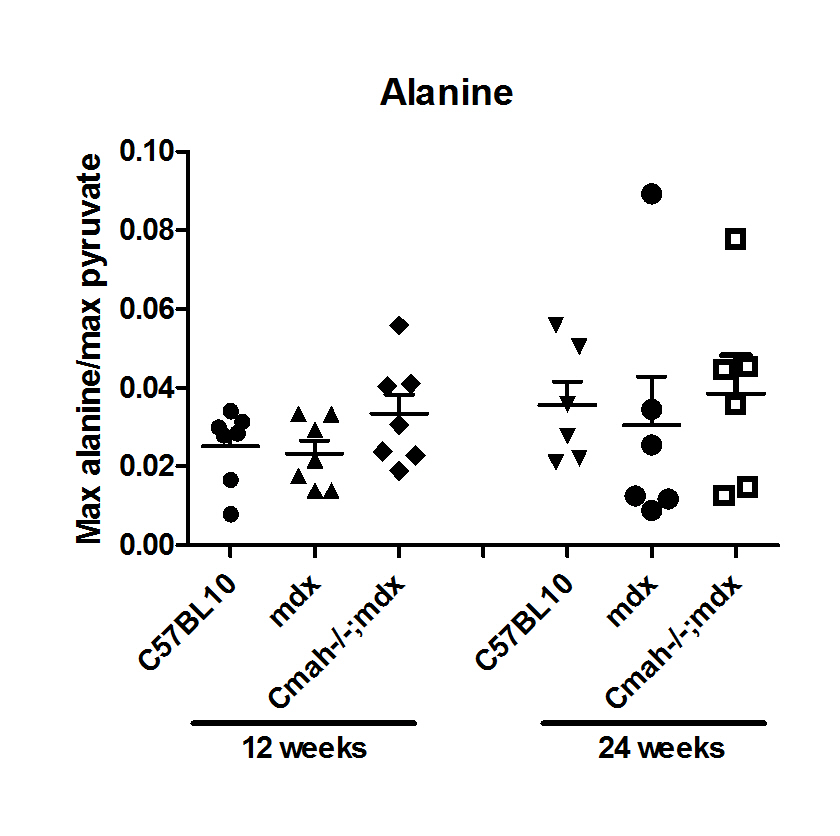
**

**Supp Figure 2. Metabolic profile of C57BL10, *mdx* and *Cmah-/-;mdx* hearts at 12 and 24 weeks of age- hyperpolarised MRS.** Alanine production normalized to maximum pyruvate signal. Data displayed as mean ± SEM. N = 5 to 7.

**
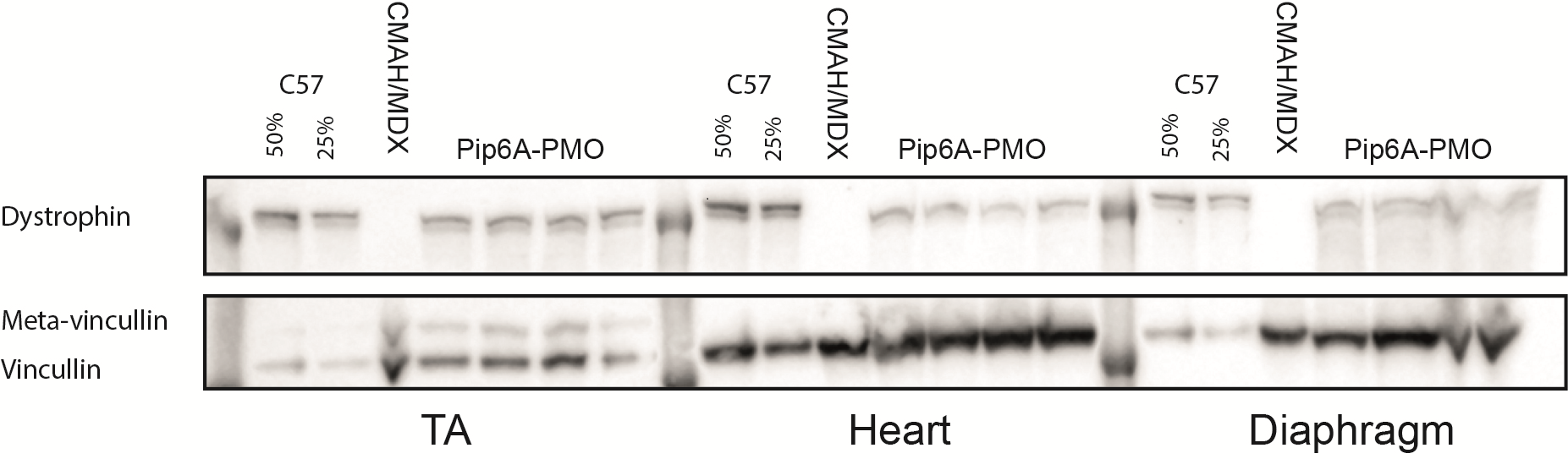
**

**Supp Figure 3.** **Dystrophin protein restoration in Pip6A-PMO *Cmah-/-;mdx* treated mice.** Dystrophin protein levels were quantified by western blotting in tibialis anterior, diaphragm and heart with normalisation to vinculin loading control. Levels were assessed against C57BL10 control tissue with 50% and 25% total protein loading.

**
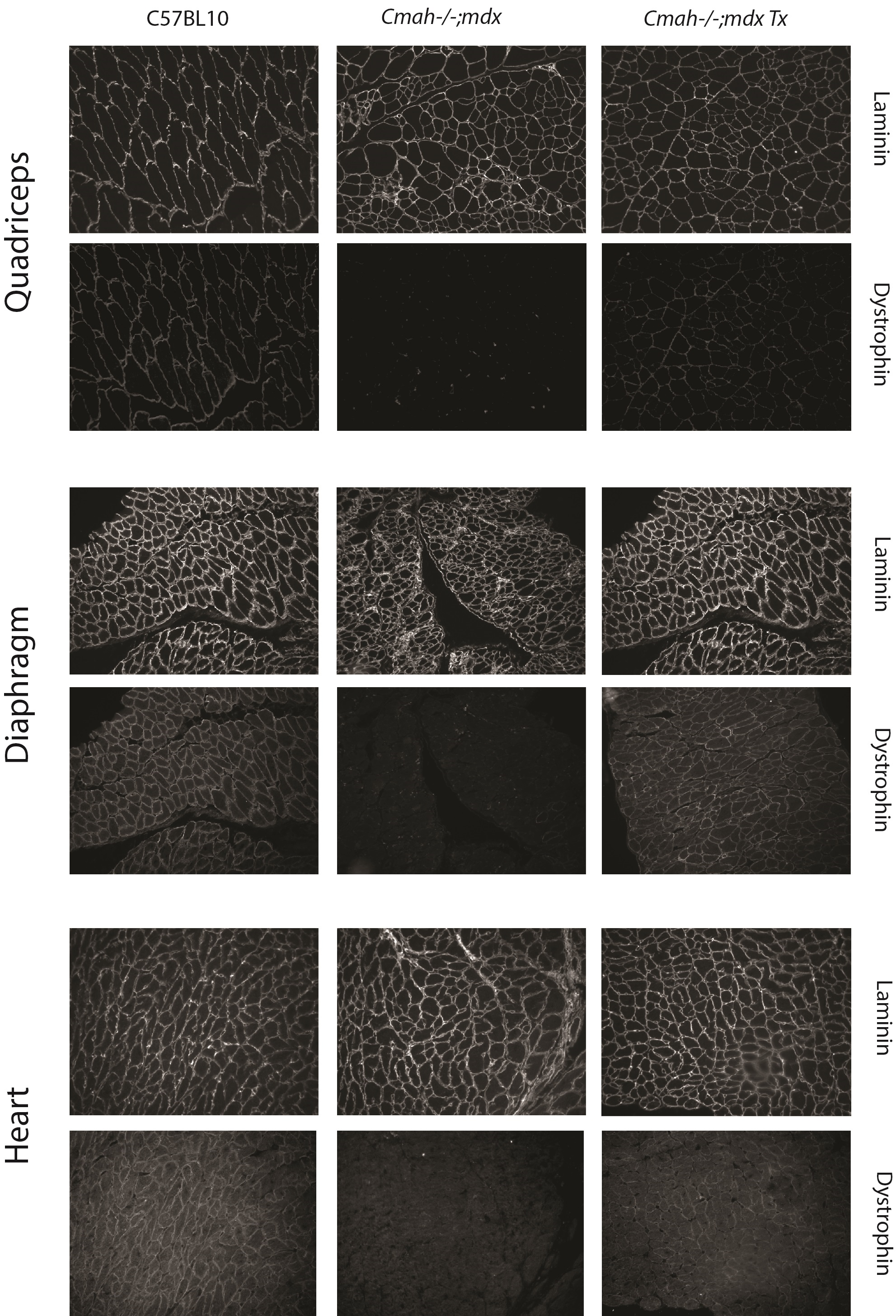
**

**Supp Figure 4.** **Dystrophin immunofluorescence in C57BL10, *Cmah-/-;mdx and* Pip6a-PMO *Cmah-/-;mdx* treated mice.** Images show dystrophin immunofluorescence in quadriceps, diaphragm and heart tissues from C57BL10, *Cmah-/-;mdx* and Pip6a-PMO *Cmah-/-;mdx* treated mice. Laminin protein immunofluorescence was used as sarcolemma staining control.


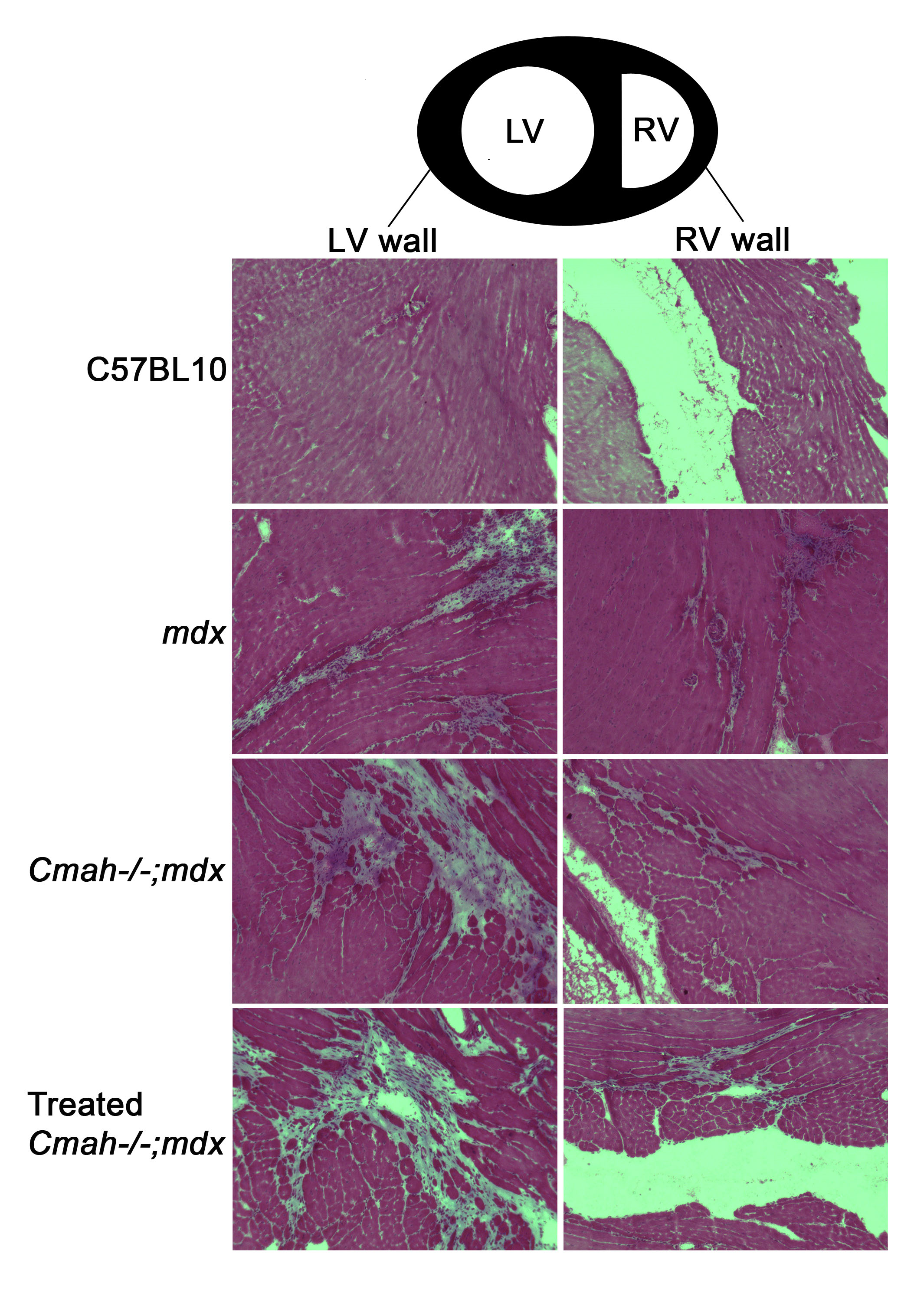


**Supp Figure 5. Masson trichrome staining of C57BL10, *mdx, Cmah-/-;mdx* and *Cmah-/-;mdx* treated hearts at 8 months of age. Representative images showing fibrosis on left ventricle (LV) wall and right ventricle (RV) wall in hearts of *mdx*, *Cmah-/-;mdx* and *Cmah-/-;mdx* treated cohorts.**

| Parameter | 12 Weeks |  |  | 24 Weeks |  |  |
| --- | --- | --- | --- | --- | --- | --- |
|  | C57BL/10 | mdx | Cmah-/-;mdx | C57BL/10 | mdx | Cmah-/-;mdx |
| ALT (U/l) | 57 ± 6 | 126 ± 10**** | 64 ± 3†††† | 37 ± 2 | 90 ± 3**** | 75 ± 5**** |
| AST (U/l) | 70 ± 6 | 657 ± 100**** | 309 ± 2***†††† | 63 ± 7 | 400 ± 10**** | 277 ± 20****† |
| LDH (U/l) | 465 ± 40 | 4222 ± 900**** | 1846 ± 100**†††† | 534 ± 50 | 2356 ± 100**** | 1518 ± 100*† |
| CK (U/l) | 864 ± 100 | 4259 ± 1000**** | 3415 ± 500*** | 604 ± 200 | 3023 ± 400**** | 1611 ± 100† |
| Amylase (U/l) | 847 ± 20 | 688 ± 30 | 546 ± 10*** | 761 ± 20 | 682 ± 20 | 699 ± 70 |
| Total cholesterol (mM) | 2.9 ± 0.1 | 3.4 ± 0.2 | 4.2 ± 0.2****†† | 3.4 ± 0.1 | 3.46 ± 0.09 | 3.9 ± 0.1*† |
| HDL (mM) | 2.02 ± 0.08 | 1.8 ± 0.2 | 2.8 ± 0.1****†††† | 2.4 ± 0.1 | 2.25 ± 0.05 | 2.55 ± 0.08† |
| LDL (mM) | 0.73 ± 0.06 | 0.87 ± 0.06 | 1.33 ± 0.06****†††† | 0.96 ± 0.02 | 1.02 ± 0.03 | 1.23 ± 0.06**† |
| Glucose (mM) | 14.4 ± 0.7 | 16 ± 2 | 13.0 ± 0.6 | 16 ± 2 | 11.1 ± 0.6** | 12 ± 1 |
| TGs (mM) | 2.1 ± 0.1 | 2.8 ± 0.4 | 2.09 ± 0.06† | 2.0 ± 0.1 | 2.5 ± 0.2 | 2.6 ± 0.2 |
| Glycerol (mM) | 378 ± 10 | 455 ± 20 | 377 ± 10 | 381 ± 40 | 348 ± 20 | 367 ± 20 |
| FFAs (mM) | 0.65 ± 0.05 | 1.0 ± 0.1** | 0.87 ± 0.04 | 0.68 ± 0.08 | 0.96 ± 0.05* | 1.12 ± 0.07*** |

**Supp Table 1. Biochemical data from plasma samples of C57BL10, *mdx* and *Cmah-/-;mdx* mice at 12 and 24 weeks.** Data displayed as mean ± SEM. N = 3 to 4 for 12 week groups. N=5 to 6 for 24 week groups. Significance calculated using two-way ANOVA, Tukey *post-hoc* test (*****P*<0.0001, ***=*P*<0.001, **=*P*<0.01*=p<0.05). *Mdx*_2 was removed from the 12 week *mdx* group for all parameters, following Grubbs’ test for outliers. ALT, alanine transaminase; AST, aspartate transaminase; LDH, lactate dehydrogenase; CK, creatine kinase; FFA, free fatty acid.

| Group | Body mass (g) | TA mass (g) | Cross sectional area (cm^2^) |
| --- | --- | --- | --- |
| C57BL10 | 33.3 (+/- 1.0) | 0.049 (+/- 0.001) | 0.069 (+/- 0.002) |
| *Cmah-/-;mdx* | 40.7 (+/- 1.3) | 0.091 (+/- 0.003) | 0.122 (+/- 0.005) |
| *Cmah-/-;mdx R_x_* | 37.1 (+/- 1.2) | 0.078 (+/- 0.003) | 0.106 (+/- 0.005) |

**Supp Table 2. Comparison of body weights, tibialis anterior (TA) weights and physiological cross-sectional area in groups undergoing muscle physiology assessment.**

| Gene Name | Assay Number |
| --- | --- |
| Ywhaz | Mm.PT.58.8991239 |
| Nppa | Mm.PT.58.8820983 |
| Nox4 | Mm.PT.58.12973594.g |
| Ctgf | Mm.PT.58.10386125.g |
| Pdk1 | Mm.PT.58.10680444 |
| Pdk4 | Mm.PT.58.9453460 |
| Ucp3 | Mm.PT.58.9090376 |
| Cpt1 | Mm.PT.58.10147164 |
| CD36 | Mm.PT.58.7548967 |
| Ppara | Mm.PT.58.9374886 |

**Supp Table 3. Integrated DNA Technologies Primer Assay ID for (qRT)-PCR.**
